# Glymphatic system, sleep, and shift work: a new paradigm in occupational and mental health?

**DOI:** 10.1590/1980-5764-DN-2022-0077

**Published:** 2023-05-05

**Authors:** Einstein Francisco Camargo, Otávio Toledo Nóbrega

**Affiliations:** 1 Universidade de Brasília, Faculdade de Medicina, Programa de Pós-Graduação em Ciências Médicas, Brasília DF, Brazil.; 2 Universidade de Brasília, Faculdade de Ciências da Saúde, Programa de Pós-Graduação em Ciências da Saúde, Brasília DF, Brazil.

Dear Editor,

October 21, 1879, was a pivotal date that changed the world. That day, Thomas Edison created a light bulb that shone for 48 h straight. After that, mankind extended the working day into the night. The economic and social gains were remarkable, with a substantial increase in the number of jobs, but causing sleep loss and circadian misalignment due to shift work. Sleep medicine, a relatively recent field of study, has made extraordinary discoveries regarding the impact of sleep on the general human health, notably on occupational and mental health.

Shift work is defined as a labor schedule that includes non-standard hours (especially evening and nighttime hours), performed constantly at a fixed schedule, at intermittent/rotating turns, or even according to less fixed time tables^1^. Changes in the natural sleep/wake cycle, also known as circadian rhythm, can trigger sleep disorders over the lifetime. There are many studies showing that sleep disorders negatively influence workers’ health, leading to conditions as hypertension, stroke, cardiovascular disease, diabetes, obesity, decreased immunity, cancer, anxiety, and depression^2^.

A recent meta-analysis assembled the findings of 18 studies encompassing 18,802 workers exposed to shift working and compared to non-exposed controls. The results showed an unquestionable impairment in cognitive performance, by means of worsened working memory, processing speed, psychomotor vigilance, cognitive control, and visual attention^3^. The authors call attention to a deteriorated performance among shift workers, including work-related injuries as well as an increased risk of clinical and procedural prescribing errors by physicians.

However, these findings go beyond a one-off risk. A population-based study revealed inverted U-shaped associations between sleep duration and subsequent cognitive decline, as well with incidence of dementia^4^. This remark was confirmed by a robust recent meta-analysis elucidating the influence of sleep disturbances on the incidence of dementia, highlighting the importance of regular sleep^5^. Results of a prospective study of two cohorts indicate that mid-life shift work history, including nighttime work, was significantly associated with increased incidence of dementia in later life^6^. Furthermore, higher dementia risk was associated with a long shift work history. In the past 10 years, new laboratory discoveries have shed light on the physiology of sleep, which may directly impact workers’ health.

A recent method for exploring and quantifying the extracellular space of the living brain (in rats) showed that deep sleep (natural or induced) increases cerebral interstitial fluid by 60%, resulting in a remarkable rise in the convective exchange between cerebrospinal fluid and interstitial fluid, which substantially increases the rate of β-amyloid protein clearance during sleep^7^. This protein is linked to Alzheimer's disease pathology. This perivascular system (named glymphatic system) clears the brain of protein waste products, being active mainly during the deepest (stage 3) phase of the non-rapid eye movement (NREM) sleep, characterized by slow-wave electroencephalogram activity^8^. Since this level of sleep tends to occur in the first half of the night, shift workers are at a higher risk for glymphatic disturbance.

Is it possible to reorganize the shift work? In modern society, more people work during “non-standard” working hours, including shift and night work. We can learn from experiences abroad. Following a growing body of evidence that children/teens have different sleep needs, hundreds of schools in developed countries have moved school start times to later hours. Now, results of a 4-year observational study carried out in a state-funded high school in England showed that changing to a 10:00 a.m. start time can significantly reduce illness and improve academic performance^9^.

Interventions to improve the health status and the working performance of healthcare professionals who undertake night shifts are of uttermost importance. Switching strict night shifts to rotating shifts may improve sleep disturbances^10^. Some integrated interventions focusing on changing individual lifestyle and working conditions have been tested^11^, but we need to go further. It is time to discuss how shift work suits in occupational medicine.

We need to understand the impact on the sleep quality from adjustments in work schedules, possibly by adding physiological data and cognitive/physical functioning tests into more comprehensive protocols of assessment. Would it be effective to introduce shorter night shifts of 4–6 h per night? Would it be favorable to reduce the workload performed at night? How about shorting the number of consecutive night shifts? How much recovery from a night shift would allow the glymphatic system to clean the brain?

Many questions remain unanswered, and researchers in mental and occupational health are invited to address these issues over the following years.
